# Biodistribution and clearance of instilled carbon nanotubes in rat lung

**DOI:** 10.1186/1743-8977-5-20

**Published:** 2008-12-09

**Authors:** Dan Elgrabli, Magali Floriani, Steve Abella-Gallart, Laurent Meunier, Christelle Gamez, Patrice Delalain, Françoise Rogerieux, Jorge Boczkowski, Ghislaine Lacroix

**Affiliations:** 1 Institut National de l'Environnement Industriel et des Risques (INERIS), Verneuil en Halatte, France; 2 Institut de Radioprotection et de Sûreté Nucléaire Cadarache (IRSN), Saint paul les durance, France; 3 Inserm, U700 ; Université Paris 7, Faculté de Médecine Denis Diderot-site Bichat, Paris, France; 4 Centre d'Investigation Clinique 007 ; Hôpital Bichat, Assistance Publique-Hôpitaux de Paris (AP-HP), Paris, France

## Abstract

**Background:**

Constituted only by carbon atoms, CNT are hydrophobic and hardly detectable in biological tissues. These properties make biokinetics and toxicology studies more complex.

**Methods:**

We propose here a method to investigate the biopersistence of CNT in organism, based on detection of nickel, a metal present in the MWCNT we investigated.

**Results and conclusion:**

Our results in rats that received MWCNT by intratracheal instillation, reveal that MWCNT can be eliminated and do not significantly cross the pulmonary barrier but are still present in lungs 6 months after a unique instillation. MWCNT structure was also showed to be chemically modified and cleaved in the lung. These results provide the first data of CNT biopersistence and clearance at 6 months after respiratory administration.

## Background

Given the low diameter of carbon nanotube (CNT), a possible translocation of this nanomaterial from the digestive tract to blood or from the lung to blood and then to other organs in the body must be considered. Inhaled ultrafine particles translocation has been previously described at low rate in a few studies with other particles like iridium or gold [1,2]. Up to now, direct investigations of CNT translocation from the lung to the blood has not been conducted. The primary reason for that is the component structure of the CNT. Only made of carbon, CNT are difficult to distinguish from biological matrix. A few studies have used indirect methods to investigate CNT biokinetics (translocation, biodistribution, clearance...). Several biokinetics studies were performed by intra-venous injection of functionalized-CNT. Wang et al (2004) analyzed the biodistribution of SWCNT using a I_125_-SWCNT construct, and revealed indirectly by radioactivity measurement of I_125_, the presence of functionalized SWCNT in stomach, kidneys and bone. Similar observations were obtained in kidneys, liver and spleen by McDevitt et al (2007) with In_111_-SWCNT. Functionalized SWCNT were found in the different organs, and biokinetics studies showed a rapid clearance of the In_111_-SWCNT from blood and tissues [3-5]. One study followed the biokinetics of injected unmodified SWCNT using inherent near-infrared fluorescence of SWCNT for detection in blood and tissue samples [6]. The authors report a rapid blood clearance and detected SWCNT only in the liver after 24 h. These results suggest a possible difference in biokinetics between modified and unmodified CNT. To investigate unmodified MWCNT biokinetics, Muller et al (2005) used another method based on the quantification of the CNT production metal catalyst residue [7]. The production of carbon nanotubes by arc discharge [8], laser ablation [9] and chemical vapor deposition [10], use metal as catalysts such as iron, nickel, cobalt, etc...which always remain as impurities in the final product [11] and can be quantified. Here, we have first assessed the performances of using the metal catalyst of CNT as a tracer. We then analyzed unmodified Nickel-catalyzed MWCNT biokinetics after intratracheal instillation.

## Results

### Analysis of metal impurities

Results showed the presence of 0.53% (w/w) of Ni, 0.08% (w/w) of S, 0.02% (w/w) of Mg, less than 0.01% (w/w) of Na and V and less than 0.005% (w/w) each for all other metals tested (listed in materials and methods).

### Analysis of Ni-CNT bonds in the lung and in the lymph nods

**Figure 1:**
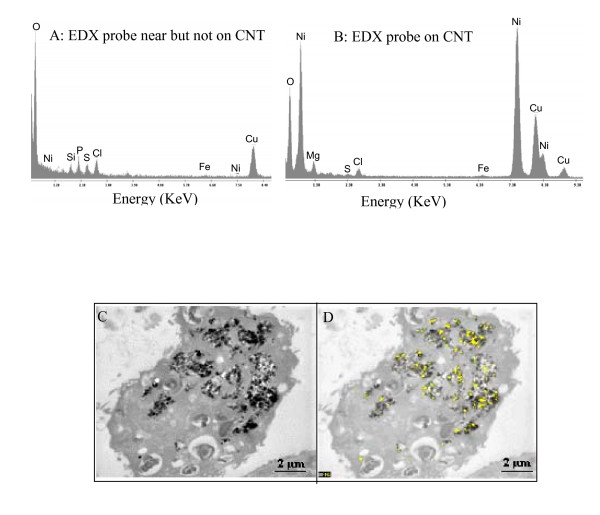
**Analysis of Ni-CNT bonds in the lung and lymph nods by STEM-EDX**. Rats were instilled by 2 mg of MWCNT. STEM-EDX analysis was performed for Ni and MWCNT detection on ultrathin sections. Spectrum of Ni analysis by EDX probe was performed during 60s in absence (A) or in presence (B) of MWCNT agglomerates. To observe the localization of Ni and MWCNT agglomerates in organism, (C) TEM photo of macrophage contains MWCNT agglomerates and (D) superposition of Ni position obtained with EDX probe (Resolution: 256 × 200; Dowell time: 800 ms; Frames: 256) in the cells were performed and reveal that MWCNT is colocalized with Ni.

STEM-EDX analysis of lungs and lymph nodes of MWCNT-exposed rats showed that peaks of Ni were observed only in the presence of MWCNT (Figure 1). The presence of Cu is due to the TEM grid used. Ni and Mg are impurities of MWCNT and additionally the last element is also normal constituent of cells. Multiple sections study of lung and lymph nodes revealed a perfect colocalization of CNT and Ni after STEM-EDX analysis (Figure 1). Ni was never detected in absence of CNT and vice-versa.

### Biokinetics studies

#### Biodistribution of MWCNT

To validate our Ni dosage method, suspensions with known final CNT quantity (0, 6.25, 12.5, 25, 50 and 100 μg) were produced in quadruplicate. The MWCNT impurity study showed that Ni was present at an average concentration of 0.53% (w/w) of total MWCNT mass. Thus, the theoretical final Ni quantities in the control suspensions are respectively 0, 0.03, 0.06, 0.12, 0.25 and 0.5 μg. All prepared suspensions were mineralized and measured Ni quantities were determined by ICP-OES as it is described in materials and methods. No statistically significance differences between theoretical and measured Ni quantities were observed (data not shown) suggesting this dosage method is relevant to determine and quantify Ni. The detection limit for this dosage was determined to be 0.5 μg/L of Ni and correspond in our experimental conditions to 100 μg/L of MWCNT.

**Figure 2:**
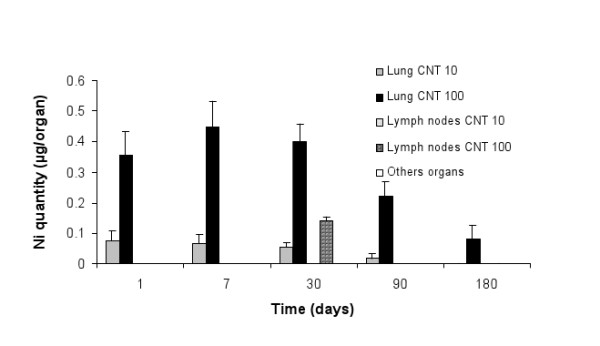
**Biodistribution of MWCNT in organism**. Presence of Ni was quantified in several organs. Representation of means ± SD of Ni quantities present in 6 rat organs from 1 to 180 days after exposure at 10 and 100 μg/rat. Results show that MWCNT doesn't cross lung barrier and is eliminated from the lung.

No increases of Ni were detectable in organs after the 1 μg or 10 μg MWCNT exposures excepted in the lung at 10 μg of MWCNT (Figure 2). No Ni was detected at any time in liver, kidneys, spleen, heart, brain, thymus and testis of rats exposed to 1, 10 or 100 μg of MWCNT. Ni was detected in the lung of MWCNT-exposed rats throughout the experiment (from 1 to 180 days) and also detected in lymph nodes at day 30 (Figure 2). Considering that Ni represent 0.53% of MWCNT instilled, the percentage of MWCNT recovered was 63% at day 1, 78% at day 7, 97% at day 30, 38% at day 90 and 16% at day 180 (Table 1). Ni was not detected in BALF at any time post-exposure (Table 1).

**Table 1:** Quantification of Ni in rat's organs after instillation of MWCNT by intratracheal instillation.

	Treatment time (days)	1	7	30	90	180
LUNG	Lung after BAL	53 ± 12%	54 ± 10%	55 ± 8%	26 ± 6%	16 ± 9%

LUNG	Alveolar cells	10 ± 2%	24 ± 5%	14 ± 2%	12 ± 2%	-

LUNG	BALF	-	-	-	-	-

	Lymph nodes	-	-	28 ± 2%	-	-

	All other organs	-	-	-	-	-

	Total	63 %	78 %	97 %	38 %	16 %

- Non Detectable (< 2% of MWCNT instillated).

Groups of 6 rats were instillated by 100 μg of MWCNT and Ni was quantified by ICP-OES. Each value represents the mean ± SD of the percentage of Ni detected related to total Ni instillated (0.5 μg).

About 50% of the instillated MWCNT were located in the parenchyma of the lung after 1 month. 10 to 24% of CNT were found in the alveolar cells during this period. At 30 days, 31% of total CNT injected was eliminated from the lung but 28% of this same nanotube was found in the lymph nods for a transition period less than 60 days. Clearance of MWCNT was amplified at 3 and 6 months after the exposure because 63% and 84% of total MWCNT injected was respectively eliminated from the organism (Table 1).

#### Clearance of MWCNT from the lung

**Figure 3:**
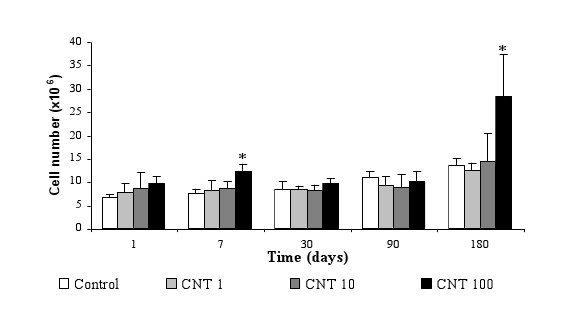
**Effect of MWCNT on alveolar cell number and on phagocytosis cells**. Rats were treated by 1, 10 or 100 μg of CNT. After BAL at d1, d7, d30, d90 and d180, total cells were quantified. Results show a significant induction of cells at d7 and d180. Results are the mean of ± SD obtained on 6 rats. * Significantly different (p < 0.05) from control group.

**Figure 4:**
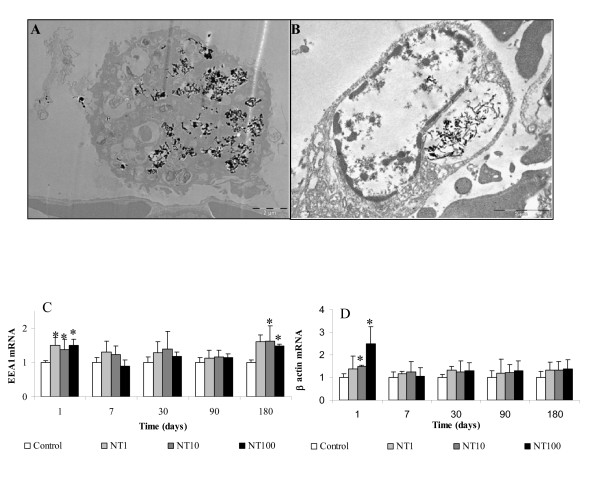
**Phagocytosis of MWCNT in vivo**. After instillation of 2 mg MWCNT, (A) lung and (B) lymph nodes sections were observed by TEM and reveal the presence of MWCNT agglomerates in macrophage vesicles. Quantification of (C) EEA1 mRNA and (D) actin beta mRNA on the lung of rats treated by 1, 10 or 100 μg MWCNT support the MWCNT phagocytosis capacity of macrophages. Results are the mean of ± SD obtained on 6 rats. * Significantly different (p < 0.05) from control group.

**Figure 5:**
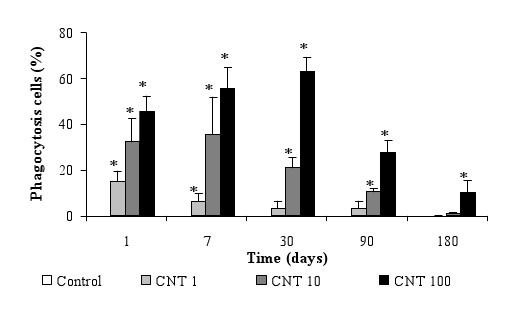
**Effect of MWCNT on alveolar phagocytosis cells**. Rats were treated by 1, 10 or 100 μg of CNT. After BAL at d1, d7, d30, d90 and d180, percentage of phagocytosis cells were quantified. Results are the mean of ± SD obtained on 6 rats. * Significantly different (p < 0.05) from control group.

To investigate the clearance mechanism, alveolar cells isolated from BAL were counted with Malassez cell and observed by optical microscopy. Significant increases in cell number, respectively plus 44% and plus 100%, were noted at 7 and 180 days after treatment (Figure 3). This variation is only due to the macrophages which represented 98% of total cells, suggesting the important role of this cell type in the bioelimination of MWCNT from the lung. Absence of variations of other cells types were noted (data not shown). TEM observations of the lung and the lymph nodes sections have shown the presence of CNT agglomerates in vacuoles (endosomes) of macrophages in these 2 organs (Figure 4A and 4B). MWCNT were never observed in parenchyma or other cell types than alveolar macrophages in the lung or in the lymph nodes. To investigate the phagocytosis capacity of lung cells, total RNA were extracted from the lung of CNT-exposed rats and specific mRNA of EEA1, an early endosome associated protein [12,13] and mRNA of β actin, a cytoskeleton protein, were quantified by RT-PCR. Results have shown plus 50% induction of EEA1 mRNA at day 1 and day 180 with all doses of CNT (Figure 4C). For β actin, induction of mRNA was significantly noted with 10 μg (plus 50% induction) and 100 μg (plus 250% induction) of CNT at day 1 (Figure 4D). Separate quantification of macrophages containing MWCNT inside their cytoplasm and macrophages without MWCNT was performed at all times and doses. Results showed a MWCNT dose-dependant increase of the number of macrophages containing agglomerates of MWCNT at all times after the exposition (Figure 5). Analysis of biopersistence of MWCNT at 1 and 10 μg, revealed a time dependent decrease of the cells number containing MWCNT agglomerates inside their cytoplasm. Nevertheless, increasing the dose of CNT induces an augmentation of the clearance time. When the rats were treated by 100 μg of CNT, 45% of their alveolar macrophages contained CNT agglomerates one day after the treatment. This percentage increased at 7 days and 1 month to 55% and 63% respectively. Diminution of the number of phagocytosing cells was only noted at 3 and 6 month (Figure 5). For lower doses, the diminution of the number of phagocytosing cells was noted at 7 days for 10 μg CNT and 1 days for 1 μg CNT.

### Physical and chemical modification of MWCNT in vivo

#### Physical modification

**Figure 6:**
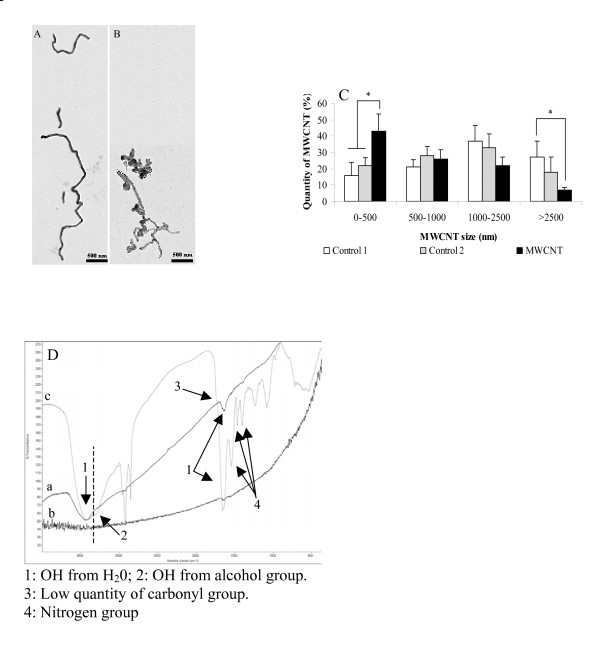
**Physical and chemical modification of MWCNT in vivo**: Physical modifications of MWCNT were observed by TEM on (A) control 2 and (B) MWCNT after intratracheal instillation. (C) Length quantification of MWCNT length was performed at least on 100 MWCNT per condition (Control1, control 2 and MWCNT after intratracheal instillation. Results are the mean of ± SD obtained on 3 independent experiment. * Significantly different (p < 0.05) from control group. (D) Chemical modifications of the MWCNT were assessed by infrared spectroscopy of (a) control 1, (b) control 2 and (c) MWCNT after intratracheal instillation. Spectra of a representative experiment performed on 3 rats.

MWCNT were instilled in rats and after 15 days, MWCNT lengths were compared to the control solutions by TEM. Results showed a modification of the structure (Figure 6A, 6B) and a significant diminution of MWCNT length suggesting the capacity of rat lung to cleave MWCNT (Figure 6C).

#### Chemical modification

After 15 days instillation of MWCNT in rat lung, chemical modifications of the MWCNT were assessed by infrared spectroscopy. Presence of alcohol, carbonyl and nitrogen function were observed on the MWCNT instillated to rat but not on the same suspension of MWCNT that was not injected in the presence or absence of cells residue (Figure 6D). Our results suggest the capacity of rat lung to chemically modify the MWCNT structure.

### Biological lung response to MWCNT

**Figure 7:**
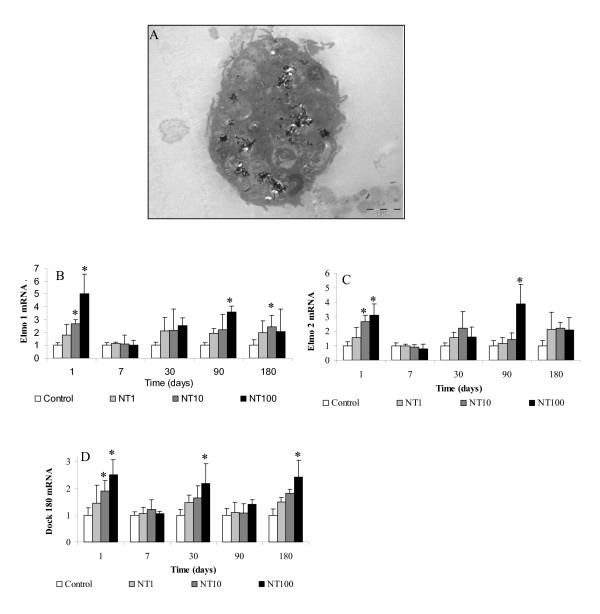
**MWCNT could induce alveolar macrophage (AM) apoptosis and apoptotic cells may be engulfed by other AM**. TEM analysis of lung sections of rat instilled with 2 mg of MWCNT showed the presence of apoptotic macrophages. This observation is supporting by the quantification of mRNA of (B) Elmo 1, (C) Elmo 2 and (D) Dock 180 suggesting the capacity of macrophages to engulf apoptotic cells. Results are the mean of ± SD obtained on 6 rats. * Significantly different (p < 0.05) from control group.

**Figure 8:**
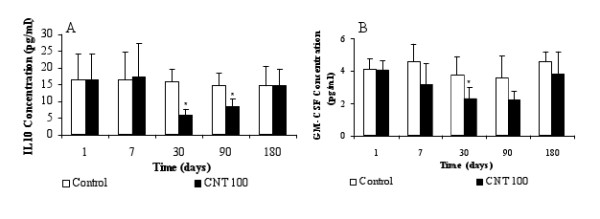
**MWCNT could inhibit the expression of IL10 and GM-CSF protein**. Rats were instilled by 100 μg of CNT and quantification of (A) IL10 and (B) GM-CSF in BALF was performed by ELISA multiplex (Luminex) after 1, 7, 30, 90, 180 days. Results are the mean of ± SD obtained on 6 rats. * Significantly different (p < 0.05) from control group.

In the lung, a fraction of cells seemed to be in apoptosis (Figure 7A) but free CNT were never found by TEM. Results have shown no modification of mRNA level for Elmo 3 protein (data not shown). But Elmo 1 protein mRNA was significantly induced 1.8, 2.7 and 5.1 times after 24 h exposure for 1, 10 and 100 μg of CNT. At day 30 and day 90, mRNA expression level was significantly increased 2.5 times with 100 μg of CNT (Figure 7B). Similar variation of mRNA level of Elmo 2 was noted because the mRNA level was induced later 1.6, 2.7 and 3.1 times at d1 for 1, 10 and 100 μf of CNT. At d90, mRNA expression level was only significantly increased 3.8 times with 100 μg of CNT (Figure 7C). Dock 180 mRNA was also induced 2.5 time 1, 30 and 180 days after the exposure (Figure 7D). Quantification of the expression of IL10 and GM-CSF which, are also involved in the engulfment of apoptotic cells, were performed. Inhibitions of 63% and 42% were noted for IL10 protein after 30 and 90 days respectively (Figure 8A). Concerning GM-CSF protein, significant inhibition was also observed 30 days after the exposure (Figure 8B).

## Discussion

Previous studies on MWCNT biodistribution were performed after tracer attachment on the surface of the tubes. But tracers modify MWCNT hydrophobicity and therefore MWCNT biodistribution. STEM-EDX analysis of our MWCNT reveals that Ni, which is an intrinsic impurity of the MWCNT we used, is strongly linked to the MWCNT and that MWCNT-Ni bonds are not affected in the body. Our results show that Ni can be used as native MWCNT tracer. More generally, utilization of metallic impurities as tracers can be a good approach to assess biokinetics of MWCNT in the body. However, the detection limit (here 0.01 μg/ml of Ni) does not allow the utilization of this method for very low concentration of MWCNT. In addition, the strength of the bond between the metal and the MWCNT can be different, depending on metals and CNT types. So that STEM-EDX bonding analysis should be performed for each CNT studied.

Ni quantification in several organs has revealed the presence of MWCNT only in the lungs and in the lymph nodes. 30 days after the instillation of MWCNT, the totality of particles was found in the lung (69%) and in the lymph nodes (28%). These results suggest no, or at least a negligible, elimination or biodistribution of CNT to other organs 1 month after instillation. Such an observation has already been done. In their study, Muller et al (2005) had found no nearly elimination of MWCNT in the rat lung 28 days after an intratracheal instillation. In our experiment, the presence of Ni in the lymph nodes was only observed at 30 days. This result can be explained by the absence of quantification between 7 days and 30 days and between 30 days and 90 days. Ni and so CNT is certainly not present in the lymph nods only 30 days after exposure but probably between more than 7 days and less than 90 days.

We have shown a decrease in the MWCNT quantity initially instilled 3 and 6 months after exposure. Three months after, only 37% of the MWCNT remained within the lungs and only 16% remained after 6 months. These observations are also in accordance with the results obtained by Muller et al, where only 18% of instilled intact MWCNT was eliminated in the lung after 60 days [7]. The fact that we failed to detect MWCNT in various systemic organs is in favor of elimination rather than a translocation of MWCNT from the lungs. Nevertheless, due to the detection limit of our Ni dosage method, a very limited translocation (< 2% of MWCNT instillated) could not ruled out. This absence of alveolar passage was also described *in vitro *on pulmonary barrier [14]. So, taken together, all these results show that MWCNT do not seem to significantly cross alveolar epithelial barrier and that they are eliminated from the lung. Previous studies on the elimination of man made mineral fibers (MMVF) showed a half clearance time near to 4 months [15]. This result suggests that MWCNT can be eliminated from the lung with an equivalent time as others fibers in the lung.

The fact that the totality of instilled MWCNT was not detected at day 1 and day 7, (respectively 63% and 78%) is probably due to the experimental protocol. Indeed, MWCNT quantification in the BALF was performed after a centrifugation troughout a membrane as described in Materials and Methods section. At this step, free MWCNT that were present at 1 and 7 days were certainly eliminated and so was not quantified. This hypothesis is strengthened by the fact that difference observed between 1, 7 and 30 days is due to an augmentation of MWCNT in alveolar cells suggesting a diminution of free MWCNT part in the lung (Table 1).

Our study of MWCNT localization in the lung showed little modification of MWCNT quantity in the parenchyma, augmentation in alveolar cells (10% at day 1 and 24% at day 7) and absence in the BALF. In parallel, the observation of the alveolar cell population by optical microscopy reveals that the cells were mainly alveolar macrophages and that there was an increase in the phagocytosing cell number well correlated with the chemical dosage of MWCNT in the cellular part of the BALF. Engulfment of MWCNT by alveolar macrophages was also confirmed by TEM observations. This cell localization for MWCNT confirms the potential capacity of alveolar macrophages to phagocyte CNT as it was described before [16,17].

**Figure 9:**
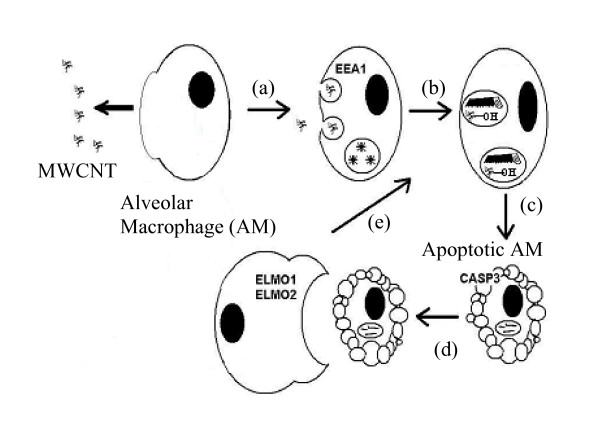
**Proposition of clearance mechanism of MWCNT in the lung**. MWCNT could be engulfed by macrophages (a), hydroxylyzed and cleaved (b). After this step, some cells enter into apoptotic phase (c). Then, a new cell could engulf the apoptotic macrophages to continue the elimination (d). The last step will be repeated as necessary to eliminate totally the MWCNT from the lung (e).

To reinforce that hypothesis, mRNA levels of EEA1 and beta actin, that are involved respectively in vesicles-endosome fusion [13] and in cytoskeleton, were quantified. 50% and 250% induction was observed for respectively EEA1 and β actin mRNA 1 day after exposure. At high magnification with TEM, cleaved fragments of MWCNT were observed in several cells. MWCNT were then extracted from lung and quantified to avoid quantification of tangled MWCNT. This result associated with the chemical modifications observed by infrared spectroscopy leads us to hypothesize that alveolar macrophages may modify the MWCNT they have engulfed, in order to facilitate their further elimination from the lung. Nevertheless, several macrophages that contain MWCNT seemed to be in apoptosis (figure 7A). This phenomena was previously described with MWCNT on primary culture of T lymphocytes and on T lymphocyte Jurkat cell line [18]. We have recently shown the capacity of this MWCNT to induce apoptosis on alveolar macrophages in vivo and in vitro [19]. The elimination of apoptotic cells needs the activation of special mechanism requiring the contribution of Elmo proteins to form a complex with DOCK180 to promote engulfment of apoptotic cells [20]. This elimination was demonstrated to be in pair with the inhibition of IL10 and GM-CSF protein and absence of inflammation [21]. We have previously shown that this MWCNT did not induce inflammation after instillation [19]. Induction of mRNA level of Elmo 1, Elmo 2 and Dock 180 associated with the absence of inflammation and inhibition of IL10 and GM-CSF protein in the lung of rat expose by MWCNT is not a proof but is in favor of the presence of apoptotic cells that are eliminated by phagocytosis. Thus, we propose the following mechanism as hypothesis to explain the MWCNT elimination from the lung (see also figure 9): MWCNT agglomerates may be first engulfed by alveolar macrophage phagocytosis. After chemical modification and cleavage of CNT, cells enter in apoptosis. Then, another macrophages could phagocyte the apoptotic cells. To eliminate all CNT, these steps could be repeated as necessary as it is shown on Figure 9.

## Conclusion

In summary, we have shown in this work that metallic impurities of CNT may be used as tracers for CNT pharmacokinetics and toxicological studies, insofar as we verified the impurities-CNT bonds. This approach reveals that the MWCNT we used don't significantly cross the pulmonary barrier and can be eliminated. This elimination might to be due in part to macrophages. This hypothesis needs further investigations to be confirmed and to reach a better understanding of the fate of CNT in the body.

## Materials and methods

### Carbon nanotubes

MWCNT (product number: 636649) was purchased from Sigma -Aldrich (Lyon, France). These nanotubes were synthesized by Chimical Vapor Deposition method. Their diameter ranged from 20 to 50 nm and their length from 0.5 to 2 μm (data from the supplier).

### Analysis of MWCNT metal impurities

MWCNT were mineralized in 5 ml nitric acid plus 5 ml chloridric acid at 180°C for 15 min in a microwave oven (MarsXpress, CEM, Matthews, NC). The mixture was filtrated and the volume was adjusted to 20 ml with distilled water. Samples were then analyzed for metal content (Li, Be, B, Na, Mg, Al, Si, S, K, Sc, Ti, V, Cr, Mn, Fe, Co, Ni, Cu, Zn, Ga, Ge, Se, Br, Rd, Sr, Y, Zr, Nb, Mo, Ru, Rh, Pd, Ag, Cd, In, Sn, Sb, Te, I, Cs, Ba, La, Ce, Pr, Nd, Sm, Eu, Gd, Tb, Dy, Ho, Er, Tm, Yb, Lu, Hf, Ta, W, Re, Os, Ir, Pt, Au, hg, Tl, Pb, Bi, Th and U) by ICP-MS (Elan, 6100DRC, Perkin Elmer- Sciex).

### Preparation of MWCNT suspensions

Different concentrations of MWCNT were suspended in NaCl in the presence of bovine serum albumin (BSA) at the same concentration as that of the MWCNT. The suspensions were sonicated 2 mn (5s pause every 10s) at 40 W with an ultrasonic probe (sonicator ultrasonic processor XL 2020, Misonix incorporated) as previously described [22]. Using this dispersion method, more than 80% of total agglomerates have a size smaller than 10 μm and correspond to breathable agglomerates [22].

### Animals

Male Sprague-Dawley rats weighing 180–220 g were purchased from Charles River Laboratories (St Germain-sur-l'Arbresle, France). The rats were kept in a conventional animal facility and had access *ad libitum *to food and drink. The experimental protocol has been approved by the local ethical committee for animal research.

### Transmission Electron Microscopy (TEM) and CNT-Nickel bond analysis by Scanning and Energy Dispersive X ray microanalysis (STEM-EDX)

2 rats were exposed to NaCl-BSA as control and 6 rats were exposed to 2 mg of MWCNT by intratracheal instillation. This high quantity was chosen in order to facilitate the detection of CNT in the lung for STEM analysis. After 7 days, the animals were anesthetized. The lungs and the lymph nodes were removed, cut in small sections and fixed with 2.5% glutaraldehyde. After a postfixation with 1% osmium tetroxide, the samples were dehydrated by ethanol and embedded in EPON 812 (TAAB). The ultrathin sections of 90 nm and 150 nm respectively for TEM and STEM-EDX analysis were obtained by an ultramicrotome (UCT, Leica), mounted on copper grids and stained with uranyl acetate and examined in a Tecnai G_2 _Biotwin (FEI) electron microscope using an accelerating voltage of 100 kV. Several photographs of entire cells and of local detailed structures were taken, analysed and compared to NaCl-BSA control samples.

To analyze the bond of CNT and nickel in the body, STEM-EDX analyses were performed. The organ sections were prepared as described above for TEM, omitting the postfixation with osmium tetroxide to avoid an Os peak in the spectra. Unstained ultrathin sections were analysed with a dispersive X-ray microanalyser equipped with a Super Ultra Thin Window (SUTW) model SAPPHIR (EDAX), at 100 kV.

### Intratracheal instillation studies

Rats were intratracheally instillated with 0, 1, 10 or 100 μg of MWCNT/rat and sacrified 1, 7, 30, 90 and 180 days later. For each concentration and for each recovery period, 2 groups of 6 rats (group A and group B) were used. Nanotube suspensions were prepared as previously described [22]. Broncho alveolar lavage (BAL) and chemical quantification of Ni were performed on rats included in the group A. Histopathology and molecular biology were assessed on rats included in the group B.

#### Nickel dosage by Inductively Coupled Plasma -Optical Emission Spectrometer (ICP-OES)

Control and exposed animals of group A were anesthetized and the lungs, liver, kidneys, spleen, heart, brain, lymph nodes, thymus and testis were removed for ICP-OES nickel assay. The lung was separated in 3 compartments: the broncho alveolar lavage fluid (BALF) for detection of free MWCNT, the cellular part of the BAL for analysis the part of MWCNT engulfed in cells and the parenchyma of the lung. The organs were mineralized in 3.5 ml of nitric acid and 1.5 ml of chloridric acid for 4 h at 100°C and then treated with 1.5 ml of H_2_O_2 _for 1 h at 95°C on a heat bloc. The volume was adjusted to 20 ml with distilled water and samples were analyzed for nickel content by ICP-OES (Ultima, HORIBA Jobin Yvon, Edison, NJ).

#### Alveolar cellularity

BAL was performed on anesthetized animals included in the group A. The lungs were washed 3-times with 9 ml of phosphate buffered saline (PBS). The BAL was centrifuged (5 min, 15wa0 g at 4°C) and the cell-free BAL (BALF) was concentrated using centrifugation (2000 g, 4°C) in Amicon Ultra tubes^® ^(Millipore) until the volume was reduced to 1 ml. The cell number was determined by counting with a Malassez cell. Differential cell count was performed on May-Grünwald-Giemsa-stained slides. Specific protein quantifications were performed in the concentrated BALF using bio-plex kit for IL10, GM-CSF (Biorad, Cat n°: 171K11070) according to the manufacturer's instructions.

#### Physical and chemical modification of MWCNT analysis by TEM and infrared spectroscopy

A 0.7 mg/ml suspension of MWCNT was produced as previously described. 150 μl of this suspension was instillated in 3 rats. After 15 days, rats were sacrified by intraperitoneal injection of pentobarbital and BAL were performed. A first centrifugation at 350 g for 10 min was performed to separate the MWCNT and the cells from the protein fraction. Cells lysis was then done in the presence of 300 μl of distillated water. After 1 h, 10 μl of this suspension was loaded on TEM copper grids to measure the length of MWCNT. In parallel, 2 types of control were realized. First control was prepared by loading directly 10 μl of the instillated suspension on TEM cooper grid. For the second control, BAL was performed on untreated rats. Cells were recovered by a 350 g centrifugation for 10 min. After removing the protein fraction, cells were lysed in the presence of water for 1 h and the suspension was put in presence of 0.7 mg of CNT. After 15 days at 37°C in oven, the suspension was centrifuged at 350 g for 10 min and resuspended in water at the same time of MWCNT instillated in rats. 10 μl of this suspension was loading on TEM cooper grid as control 2.

Treated MWCNT, control 1 and control 2 suspensions were then centrifuged at 350 g for 10 min. Supernatant was eliminated by pipeting and put at 37°C over night to obtain a dry powder. Chemical modification was then assessed by infrared spectroscopy (Nicolet 510M) on 70 μg of commercial MWCNT powder, control 1 powder, control 2 powder and treated MWCNT in the presence of 260 μg of KBr.

#### Phagocytosis makers' by reverse transcription real time polymerase chain reaction (RT-qPCR)

30 mg of lungs from group B rats was disrupted using Precellys^® ^24 lysis/homogenizer (Bertin technologies, France) and total RNA was extracted with RNeasy^® ^plus Mini Kit according to manufacturer's protocol in presence of 600 μl of RLT buffer. 1 μg of total RNA was reverse transcribed to cDNA with Omniscript^® ^Reverse Transcription kit (Qiagen, France) according to the manufacture's protocol using OligodT primer (Qiagen).

The quantification of mRNA for RPL32 (first housekeeping gene, Cat n° QT00444857), GNB2L1 (second housekeeping gene, Cat n° QT00193872), EEA1 (Cat n° QT01619121), β actin (Cat n° QT00193473), Elmo 1 (Cat n° QT01581930), Elmo 2 (Cat n° QT00365680), Elmo 3 (Cat n° QT00382214) and Dock 180 (Cat n° QT01684081), transcript were performed using QuantiTect™ Primer^® ^Assay (Qiagen) and QuantiTect™ Sybr Green^® ^Kit (Qiagen) according to the manufacturer's protocols on 100 ng of cDNA using Mastercycler^® ^ep *realplex 4 *S (eppendorf, France). Each sample was run in triplicate.

### Statistics

All data were expressed as mean ± S.D (standard deviation). Differences between groups were assessed by the one-way analysis of variance (ANOVA). If the variances between groups were homogenous, groups were subjected to the multiple comparison Dunnett's test. If the variances were not homogeneous, groups were compared by the Mann-Whitney test. *P < 0.05 was considered as the statistical significance level.

## Competing interests

The authors declare that they have no competing interests.

## Authors' contributions

All authors read and approved the final manuscript.
